# Spontaneous Coronary Artery Dissection in a Patient with Undiagnosed Ehlers-Danlos Syndrome

**DOI:** 10.7759/cureus.4065

**Published:** 2019-02-13

**Authors:** Zeid Nesheiwat, Muhammad A Mangi, Daniel Kosinski

**Affiliations:** 1 Cardiology, University of Toledo Medical Center, Toledo, USA

**Keywords:** spontaneous coronary artery dissection, myocardial infarction, ehlers-danlos syndrome, obtuse marginal artery, circumflex coronary artery, scad, connective tissue disease

## Abstract

Spontaneous coronary artery dissection (SCAD) is a rare and deadly cause of acute myocardial infarction (MI). It remains greatly misdiagnosed and carries a high in-hospital mortality rate. Herein, we report a healthy 38-year-old female who presented to our institution for non-ST segment myocardial infarction, in which subsequent coronary angiogram revealed a type 2 spontaneous coronary artery dissection of the obtuse marginal branch with diffuse single-vessel disease of the circumflex artery. After a thorough evaluation, the patient was found to have underlying Ehlers-Danlos Syndrome that was previously undiagnosed. The patient was medically treated with dual antiplatelet therapy and statin and discharged with strict follow-up. This case is a good example of a rare and life-threatening disease process that was observed in a patient who was found to have Ehlers-Danlos Syndrome that was previously unknown.

## Introduction

Spontaneous coronary artery dissection (SCAD) remains a deadly and life-threatening cause of myocardial infarction (MI) and in some cases, sudden cardiac death. SCAD is commonly associated with typical signs of acute coronary syndrome (ACS); however, the underlying mechanism is non-traumatic, non-iatrogenic, and non-thrombotic. It is caused by a disruption of a coronary vessel wall leading to a false lumen and hence impaired blood flow causing myocardial ischemia. SCAD is estimated to be the culprit in 0.5% of cases of acute myocardial infarction [1]. SCAD accounts for approximately 25% of MI in women less than 50 years of age [2]. Risk factors predisposing to SCAD include female sex, especially in the peripartum state, hormone therapy, connective tissue disorders (i.e. fibromuscular dysplasia, Ehlers-Danlos syndrome, and Marfan syndrome), and systemic inflammatory conditions. It has been reported that approximately 5% of cases are associated with connective tissue disease [3]. Precipitating stressors that may provoke SCAD in vulnerable patients include illicit drug use, exercise, and Valsalva-type maneuvers such as labor. Coronary angiogram is the gold standard for diagnosis but if inconclusive, intracoronary imaging with optical coherence tomography (OCT) or intravascular ultrasound (IVUS) may be helpful. The management of SCAD varies, but conservative management is the preferred method. Herein, we present a 38-year-old woman who presented with worsening chest pain and an elevated troponin level. Coronary angiography showed spontaneous dissection of the left obtuse marginal (OM) artery with isolated diffuse single-vessel disease of the left circumflex artery. The patient was discharged with strict medical management and early follow-up in the outpatient clinic.

## Case presentation

**Figure 1:**
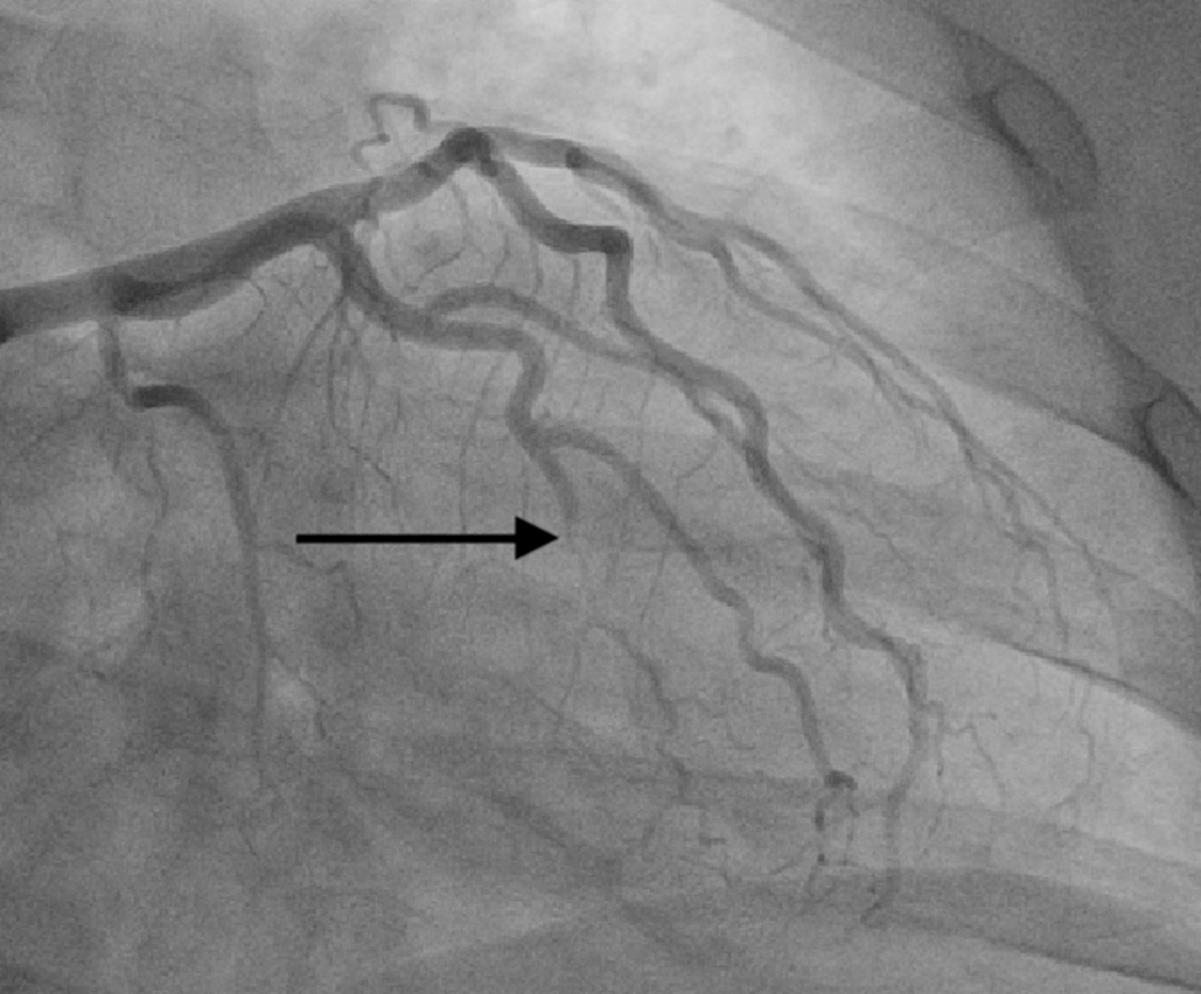
The black arrow demonstrates a type 2 spontaneous coronary artery dissection of the obtuse marginal branch of the circumflex artery

**Figure 2:**
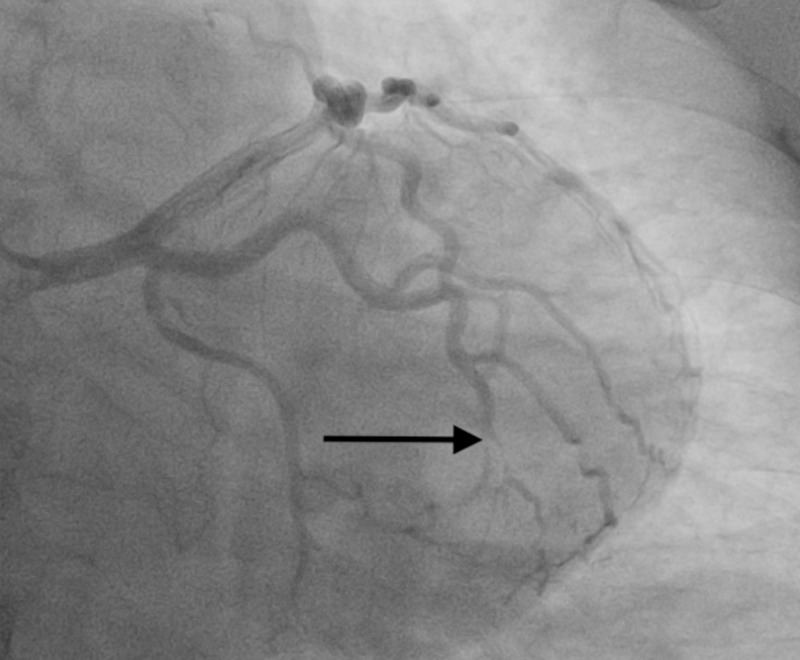
The black arrow demonstrates a type 2 spontaneous coronary artery dissection of the obtuse marginal branch of the circumflex artery

A 38-year-old female with a past medical history of hypothyroidism, hypertension, and tobacco use presented to our emergency department with a chief complaint of intermittent burning-like retrosternal chest pain that has been occurring for the past three days but has significantly worsened over one day. In the emergency department, the patient was hemodynamically stable but symptomatic. Electrocardiogram revealed normal sinus rhythm without evidence of ST or T wave changes; however, initial troponin levels were elevated. Due to the patients' sedentary lifestyle, CT angiogram of the chest was done to rule out pulmonary embolism. Subsequently, cardiology was consulted and the decision to pursue cardiac catheterization was made for continued rising troponin levels and persistent chest pain. Cardiac catheterization revealed a SCAD of the OM branch of the left circumflex artery (Figures 1, 2). In addition, there was isolated narrowing and diffuse vessel disease of the circumflex artery without atherosclerotic disease elsewhere. Percutaneous coronary intervention (PCI) was not performed as SCAD involved small and distal vessels, hence; medical management was initiated with dual antiplatelet therapy along with strict risk modification. During her hospital stay, further inquiry and thorough workup confirmed the diagnosis of Ehlers-Danlos syndrome.

## Discussion

SCAD is a rare and life-threatening cause of MI. Although the underlying mechanism is not fully understood, damage to the vasa vasorum with intramedial hemorrhage has been theorized [4]. This results in the creation of a false lumen with the development of an intramural hematoma and pressure-driven expansion leading to luminal collapse [5]. Changes in hemodynamics and various hormonal effects have been proposed in peripartum females who develop SCAD [6]. Clinical manifestations are typical of ACS with chest pain presenting in 96% of cases [7]. The left anterior descending artery (LAD) is the most frequently involved vessel showing up in about 40% of cases [7-8]. However, dissection of the OM arteries has been reported in 15% to 40% of cases [9]. It is important to note that proximal vessel dissection is rare, occurring in less than 10% and multivessel involvement occurring in 9% to 23% of cases [9]. The diagnosis of SCAD is designated into three main types; type 1 is described when a double lumen is identified in the affected vessel, type 2 is defined as diffuse narrowing of the affected vessel and is the most common, occurring in 67% of cases, and type 3 is when the affected lesion mimics atherosclerotic disease. Type 3 is often missed, and therefore, the true prevalence of SCAD may be underestimated [10]. The diagnosis is typically made with a coronary angiogram, but OCT and IVUS may be used if coronary angiogram is inconclusive. Some physicians suggest a repeat coronary angiogram within four to six weeks to evaluate vessel healing. Our patient had type 2 SCAD involving the OM branch of the left circumflex artery.

Conservative therapy with dual antiplatelet therapy, beta-blocker, and statin (in patients with dyslipidemia) is recommended, but the optimal treatment remains uncertain [3]. PCI has been a widely acceptable method of management in certain cases but remains challenging due to vessel wall fragility. In rare cases, coronary bypass and fibrinolytic therapy have been indicated and reported. In our case, the patient was found to have isolated diffuse single-vessel disease of the circumflex with spontaneous dissection of the OM; hence, we opted for medical management with close early follow-up in the outpatient clinic. Prognosis for SCAD is varied. In the largest cohort study, in-hospital mortality was 4.2% in patients treated conservatively [1]. The recurrent in-hospital MI rate was 4.6%, with unplanned revascularization in 4.3% of patients. Long-term adverse events are common with SCAD occurring within 3.1 years; MI occurred in 16.8% of which, 10.4% were recurrent SCAD [8]. Upon discharge, it is important to consider cardiac rehabilitation, and long-term inter-professional care should encompass cardiovascular health education, dietary counseling, psychosocial support, and exercise rehabilitation. It is important to note that patients who develop SCAD should be advised to avoid heavy lifting, and exercise should be targeted to a heart rate no more than 50% to 70% of heart rate reserve and maximum systolic blood pressure of <130 mmHg during “moderate” to “somewhat difficult” exercise [11]. In conclusion, SCAD remains a greatly underdiagnosed cause of MI, and proper precautions should be taken to prevent recurrence. 

## Conclusions

SCAD is a non-atherosclerotic and non-iatrogenic cause of acute MI. Although rare, every medical professional should have this diagnosis in mind when younger patients, peripartum patients, and patient with systemic or connective tissue disease present with symptoms of ACS due to its potentially fatal outcomes. Our case is a unique example of a younger patient with a previous history of undiagnosed Ehlers-Danlos Syndrome who was found to have a SCAD of the OM artery. Overall, SCAD presents in 0.5% of MI, of which, our case involved the OM artery, which is present in less than a third of cases. With an in-hospital mortality rate of about 5% and a recurrence rate of up to 20%, strict follow-up and risk modification therapy are the forefront of treatment and prevention in high-risk patients.
